# Supplementary material for: Wearable Capacitive Pressure Sensor for Contact and Non-Contact Sensing and Pulse Waveform Monitoring
Source: Molecules. 2022 Oct 13;27(20):6872. doi: 10.3390/molecules27206872 (PMC9608491; doi:10.3390/molecules27206872)
Supplement: Supplementary file 1 [file molecules-27-06872-s001.zip › molecules-1902137-supplementary.pdf]

Supporting Information

Wearable Capacitive Pressure Sensor for Contact and Non-Contact Sensing and Pulse Waveforms Monitoring

Azmal Huda Chowdhury [1], Borzooye Jafarizadeh [1], Nezih Pala [2] and Chunlei Wang [1,3,*]

[1] Department of Mechanical and Materials Engineering, Florida International University, Miami, FL 33174, USA
[2] Department of Electrical and Computer Engineering, Florida International University, Miami, FL 33174, USA
[3] Center for Study of Matter at Extreme Conditions, Florida International University, Miami, FL 33174, USA
* Correspondence: wangc@fiu.edu

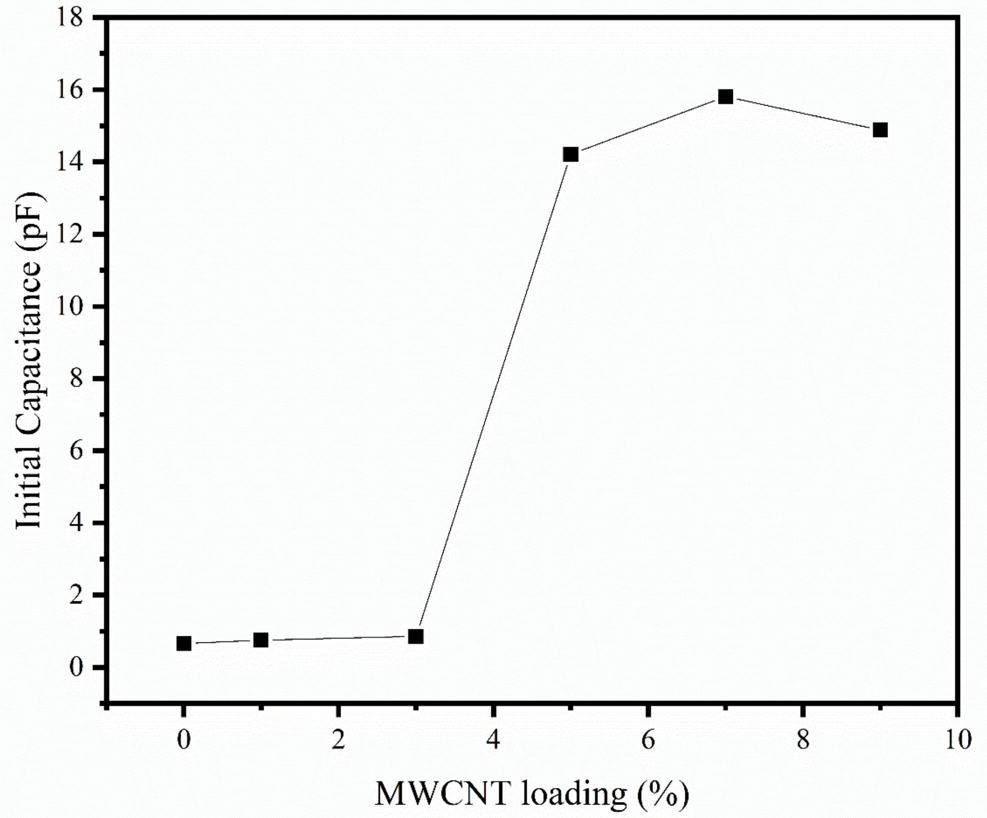

Figure S1: Change in base capacitance with the addition of MWCNT.

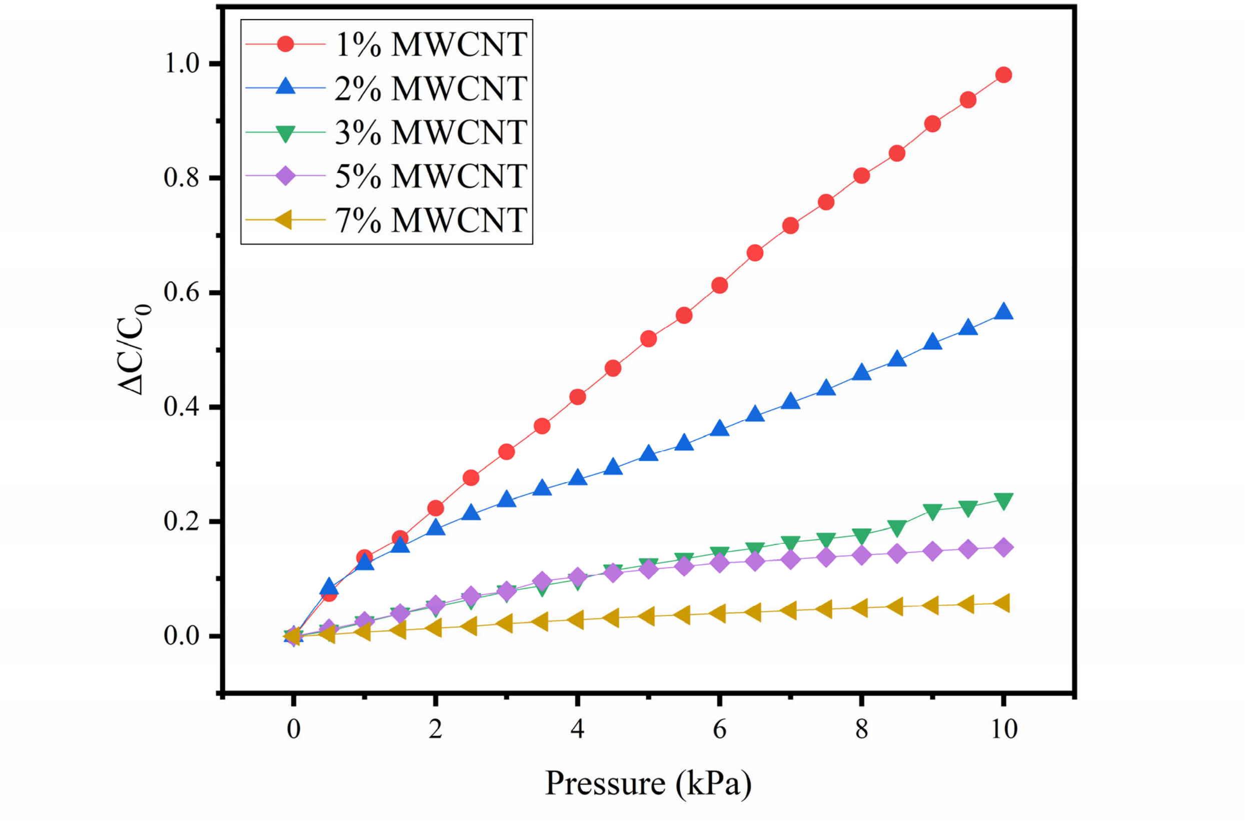

Figure S2: Decrease in relative capacitance with higher concentration of MWCNT.

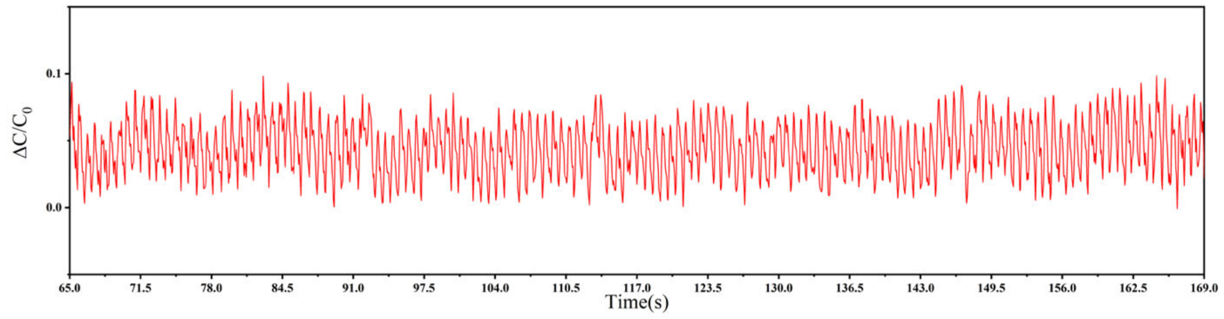

Figure S3: Pulse waveforms collection for a wide range of data collection.

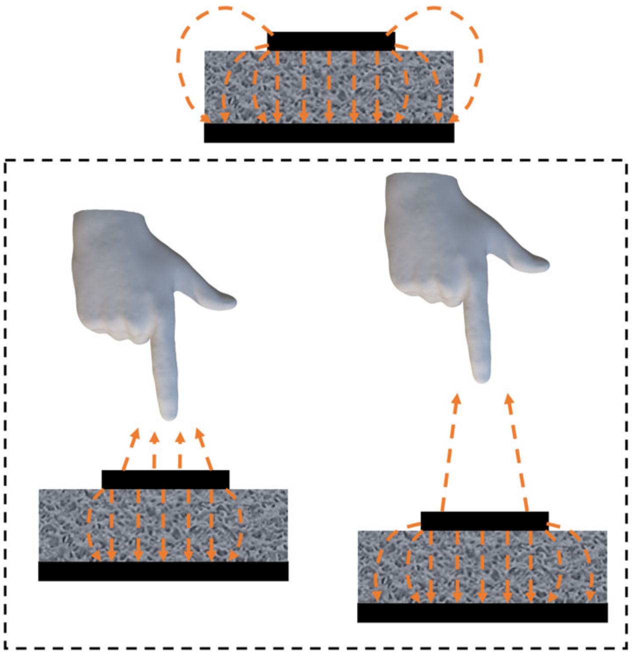

Figure S4: Proximity sensing mechanism
